# Two mycenoid species of *Leucoinocybe* (Agaricales, Basidiomycota) from southern China

**DOI:** 10.3897/mycokeys.129.174720

**Published:** 2026-03-12

**Authors:** Wen-Xiao Xia, Chao-Qun Wang, Xiang-Lian Chen, Rui-Hua Sun, Cun-Xiang Fan, Tai-Hui Li, Wang-Qiu Deng, Hai-Mei Yue, Ming Zhang

**Affiliations:** 1 State Key Laboratory of Applied Microbiology Southern China, Guangdong Provincial Key Laboratory of Microbial Culture, Collection and Application, Institute of Microbiology, Guangdong Academy of Sciences, Guangzhou 510070, China Institute of Microbiology, Guangdong Academy of Sciences Guangzhou China; 2 College of Plant Science, Xizang Agricultural and Animal Husbandry University, Linzhi 860000, China Guangdong University of Education Guangzhou China; 3 College of Biology and Food Engineering, Guangdong University of Education, Guangzhou 510303, China Hunan University of Medicine Huaihua China; 4 School of Medical Laboratory Science, Hunan University of Medicine, Huaihua 418000, China Xizang Agricultural and Animal Husbandry University Linzhi China; 5 Haizhu National Wetland Park, Guangzhou 510000, China Haizhu National Wetland Park Guangzhou China

**Keywords:** Mycenoid, new taxon, phylogeny, Porotheleaceae, taxonomy

## Abstract

Two new species of *Leucoinocybe* from southern China are described, illustrated, and compared with phenotypically similar and phylogenetically related species. Morphologically, *L. danxiashanensis***sp. nov**. is characterized by its mycenoid basidiomata, light brown to brownish orange pileus with faintly yellow striations, decurrent lamellae, abundant lageniform to obclavate and thin-walled cystidia on pileus and stipe surface, and broadly ellipsoid to subamygdaliform basidiospores; and *L. haizhuensis***sp. nov**. is characterized by its mycenoid basidiomata, brown, light brown to yellowish white pileus with pubescent, 2-spored basidia, ovoid, diverse cheilocystidia and elliptical to broadly elliptical basidiospores. Phylogenetic analyses based on sequences of the nuclear ribosomal large subunit (nrLSU) and the nuclear ribosomal internal transcribed spacer (nrITS) supported them as two distinct species of *Leucoinocybe*.

## Introduction

*Leucoinocybe* Singer ex Antonín, Borovička, Holec & Kolařík, typified by *L. lenta* (Maire) Antonín, Borov., Holec & Kolařík, is a recently established agaricoid genus according to the provisional name “*Leuco-Inocybe*” which was proposed by Singer (1943) (Antonín et al. 2019). *Leucoinocybe* species are mainly characterized by their smooth and amyloid basidiospores, various cheilocystidia, cutis pileipellis, inamyloid parallel lamellar trama, thin to thick-walled pileocystidia and caulocystidia (Antonín et al. 2019; Kaygusuz et al. 2020; Na et al. 2021, 2024). Before the establishment of *Leucoinocybe*, species with those characteristics mentioned above were placed in *Clitocybula* (Singer) Singer ex Métrod (Métrod 1952; Bigelow 1973; Vila 2002; Vigrós et al. 2005; Deepna Latha et al. 2015). Phylogenetic analyses showed that *Leucoinocybe* formed a monophyletic genus level clade and nested within the family Porotheleaceae Murrill (Antonín et al. 2019; Vizzini et al. 2019).

To date, eight *Leucoinocybe* species have been reported worldwide (www.indexfungorum.com, accessed on Jan 7, 2026). They are widely distributed in Africa, Asia and Europe, and mainly grow on bark, rotten wood and underground roots of living grasses (Takahashi 1999; Vila 2002; Contu 2003; Malysheva et al. 2011; Deepna Latha et al. 2015; Antonín et al. 2019; Na et al. 2021, 2024; Cazabonne et al. 2025; Yang et al. 2025). In China, only four species have been reported, and mainly distributed in the subtropical to tropical regions of southern China (Na 2019; Na et al. 2021, 2024; Yang et al. 2025).

During our survey of fungal diversity in southern China, several samples of *Leucoinocybe* were collected from Guangdong Province. Further study proved that they represent two species new to science, which are formally described below.

## Materials and methods

### Sample collection and morphological study

Fresh specimens were annotated, photographed, and observed in the field when collected, then dried in an electric drier, and lastly deposited at the Fungarium of Guangdong Institute of Microbiology (GDGM). Colors were confirmed and described according to Kornerup and Wanscher (1978). Microscopic methods followed Xia et al. (2025). Microscopic structures were observed by using thin, free-hand sections mounted in 5% KOH solution, 1% Congo Red solution and Melzer’s reagent under a light microscope. For each collection, 30 basidiospores and 10 elements of each of the following structures were measured: basidia, cheilocystidia, pileocystidia, caulocystidia, hyphae of the lamellar trama, pileipellis, and stipitipellis.

The notations “basidiospores (n/m/p)” indicate that the measurements were made on ‘n’ basidiospores from ‘m’ basidiomata of ‘p’ collections. Dimensions of basidiospores are presented in the following form (a)b–c(d), in which ‘b–c’ contains 90% of the measured values and extreme values ‘a’ and ‘d’ are set in parentheses, each extreme represents 5%. L_m_ = L ± SD and W_m_ = W ± SD, where L and W denote basidiospore length and width, respectively; Q_m_ = Q ± SD, Q = L/W, where SD represents the standard deviation. All microscopic features were drawn freehand.

### DNA extraction, PCR amplification and sequencing

Genomic DNA was extracted from a silica gel-dried specimen using the Sangon Fungus Genomic DNA Extraction kit (Sangon Biotech Co., Ltd., Shanghai, China) complying with the manufacturer’s instructions. The regions of nrITS and nrLSU were amplified using the primers ITS1/ITS4 and LR0R/LR5, separately (Vilgalys and Hester 1990; White et al. 1990). PCR was performed in a total volume of 25 μL containing 1 μL template DNA, 1 μL of each primer, 9.5 μL ddH_2_O, and 12.5 μL PCR mix. PCR and cycle sequencing reactions followed standard protocols; the reaction conditions were 35 cycles of 94 °C for 30 s, 55 °C for 40 s and 72 °C for 70 s, followed by a final extension at 72 °C for 8 min. Amplified PCR products were purified and sequenced by Guangzhou Tianyi Huiyuan Gene Technology Co., LTD. After that, the raw DNA sequence files were edited and assembled with Geneious Prime (https://www.geneious.com). The edited sequences were then used for a BLAST search in GenBank (https://blast.ncbi.nlm.nih.gov) to make sure the sequences were not contaminated.

### Phylogenetic analyses

Two datasets were assembled in this study. Dataset I (nrITS-LSU) was used to infer the intergeneric placement of the newly discovered species in China within the Porotheleaceae. The representative species sequences of 15 genera of Porotheleaceae were selected from the GenBank database referring to Na et al. (2024) and Yang et al. (2025). And sequences of *Mycena purpureofusca* (Peck) Sacc. were selected as the outgroup following Na et al. (2024). Dataset II (nrITS) was used to infer the interspecific phylogenetic relationships between the new Chinese *Leucoinocybe* species and the other species of *Leucoinocybe*, because GenBank contains a large amount of nrITS sequence data from the genus.

Individual datasets were aligned separately using MAFFT v7.505 with the auto strategy (Katoh and Standley 2013), and manually modified in Geneious Prime (https://www.geneious.com). The nrITS and nrLSU alignments were concatenated using Concatenate Sequence within PhyloSuite v1.2.3 (Zhang et al. 2020; Xiang et al. 2023), and treated as an nrITS-LSU dataset for further phylogenetic analyses. Optimal models for Maximum Likelihood (ML) and Bayesian Inference (BI) analyses were determined using ModelFinder v2.2.0 (Kalyaanamoorthy et al. 2017). ML analysis was performed using IQ-TREE implemented in PhyloSuite v1.2.3, with node support assessed via 5000 ultra-fast bootstrap replicates (Minh et al. 2013; Nguyen et al. 2015; Zhang et al. 2020; Xiang et al. 2023). BI analysis was conducted using MrBayes v3.2.7a within the same platform to evaluate posterior probabilities (PP) (Ronquist et al. 2012). The BI analysis ran for 5 million generations with a Sampling frequency of 1000, 2 runs, 4 chains; the initial 25% of sampled data were excluded as burn-in, and final Average standard deviation of split frequencies (ASDSF) under 0.01 (Ronquist et al. 2012). Phylogenetic trees were visualized using FigTree v1.4.4 (Rambaut 2018).

## Results

### Molecular phylogenetic results

Seventeen newly generated nrITS and nrLSU sequences have been deposited in GenBank with accession numbers (Table 1). Sixty-six nrITS sequences and forty-five nrLSU sequences of 15 genera of Porotheleaceae were downloaded from GenBank database. These downloaded sequences combined with newly generated ones were used for phylogenetic analyses. The final nrITS dataset consisted of 75 sequences, and the final nrITS-LSU concatenated dataset included 75 nrITS and 53 nrLSU sequences.

**Table 1.** Taxa, voucher numbers, localities, GenBank accession numbers and references of samples used in phylogenetic analyses are provided. Holotypes are indicated as HT, and epitypes as ET. Newly generated sequences are presented in bold.

Taxon	Voucher number	Locality	GenBank accession numbers		Reference
			nrITS	28S nrLSU	
*Chrysomycena perplexa*	MCVE:30184 (HT)	Italy	NR172974	NG071251	Vizzini et al. (2019)
*Clitocybula fuscostriata*	FFAAS1029	China	OR238881	OR238893	Na et al. 2024
*Clitocybula fuscostriata*	FFAAS1030 (HT)	China	OR238882	OR238894	Na et al. 2024
*Clitocybula fuscostriata*	FFAAS1031	China	OR238883	OR238895	Na et al. 2024
*Delicatula integrella*	KA12-1305	South Korea	KR673538	-	Kim et al. (2015)
*Delicatula integrella*	S.D.Russell MycoMap # 6067	USA	MN905231	-	Direct Submission
*Gerronema baishanzuense*	FFAAS0359 (HT)	China	OL985962	OL985984	Na et al. (2022)
*Gerronema pubescens*	GDGM 93936	China	PQ452700	PQ350414	Zhang et al. (2025)
*Hydropodia subalpina*	OKA-TR-K364	Turkey	MN701620	MN700170	Direct Submission
*Hydropodia subalpina*	STU:SMNS-STU-F-0900123	Germany	MF039248	-	Direct Submission
*Hydropus fuliginarius*	S.D. Russell ONT iNaturalist # 130794969	USA	OP643427	-	Direct Submission
*Hydropus marginellus*	PBM2344-WTU	USA	DQ490627	DQ457674	Matheny et al. (2006)
*Hydropus rugosodiscus*	TENN:070547	USA	KY777390	-	Direct Submission
*Leucoinocybe auricoma*	HKAS 41510	China	DQ490647	DQ470812	Matheny et al. (2006)
*Leucoinocybe auricoma*	HKAS126433	China	OQ025169	-	Direct Submission
***Leucoinocybe danxiashanensis***	GDGM79543	China	MZ667475	MZ667479	This study
***Leucoinocybe danxiashanensis***	GDGM80113	China	MZ667476	MZ667480	This study
***Leucoinocybe danxiashanensis***	GDGM80114 (HT)	China	MZ667477	MZ667481	This study
***Leucoinocybe danxiashanensis***	GDGM80184	China	MZ667478	MZ667482	This study
*Leucoinocybe flavoaurantia*	D	Italy	HM191743	-	Malysheva et al. (2011)
*Leucoinocybe flavoaurantia*	GDOR	Italy	HM191744	-	Malysheva et al. (2011)
*Leucoinocybe flavoaurantia*	LE 262757	Italy	HM191745	-	Malysheva et al. (2011)
***Leucoinocybe haizhuensis***	GDGM94606	China	PX401568	PX401573	This study
***Leucoinocybe haizhuensis***	GDGM95340	China	PX401569	-	This study
***Leucoinocybe haizhuensis***	GDGM99242	China	PX401570	PX401574	This study
***Leucoinocybe haizhuensis***	GDGM99420	China	PX401571	PX401575	This study
***Leucoinocybe haizhuensis***	GDGM102360 (HT)	China	PX401572	PX401576	This study
*Leucoinocybe lenta*	OKA-TR-M427	Turkey	MN701623	MN700173	Kaygusuz et al. (2020)
*Leucoinocybe lenta*	OKA-TR-M498	Turkey	MN701624	MN700174	Kaygusuz et al. (2020)
*Leucoinocybe lenta*	OKA-TR-M500	Turkey	MN701625	MN700175	Kaygusuz et al. (2020)
*Leucoinocybe lenta*	AMB 18837	Italy	OM422765	OM423643	Consiglio et al. (2022)
*Leucoinocybe lenta*	BOZ (ET)	Italy	PV367406	LT854032	Antonín et al. (2019)
*Leucoinocybe lenta*	ERD-9928	Spain	PQ587292	-	Antonín et al. (2019)
*Leucoinocybe lishuiensis*	FFAAS0111 (HT)	China	MW424488	MW424492	Na et al. (2021)
*Leucoinocybe lishuiensis*	FFAAS0112	China	MW424489	MW424493	Na et al. (2021)
*Leucoinocybe lishuiensis*	FFAAS0113	China	MW424490	MW424494	Na et al. (2021)
*Leucoinocybe lishuiensis*	FFAAS0115	China	MW424491	MW424495	Na et al. (2021)
*Leucoinocybe papillata*	TRTC176854 (HT)	Pakistan	OP497986	OP497985	Cazabonne et al. (2025)
*Leucoinocybe papillata*	TRTC176855	Pakistan	OP497984	OP497987	Cazabonne et al. (2025)
*Leucoinocybe papillata*	LAH37831	Pakistan	OQ998346	-	Direct Submission
*Leucoinocybe parviauricoma*	HKAS150759 (HT)	China	PX308922	PX309040	Yang et al. (2025)
*Leucoinocybe* sp.	WQGY2021-6-14	China	OK586755	OK586694	Antonín et al. (2019)
*Leucoinocybe* sp.	JLF13318 AZ iNaturalist # 251572772	USA	PQ666439	-	Direct Submission
*Leucoinocybe* sp.	FDS-CA-01380	USA	PQ160956	-	Direct Submission
*Leucoinocybe* sp.	FDS-CA-01829	USA	PV577611	-	Direct Submission
*Leucoinocybe* sp.	HAY-F-001811	USA	OR860219	-	Direct Submission
*Leucoinocybe subglobispora*	FFAAS1034 (HT)	China	OR238886	OR238898	Na et al. (2024)
*Leucoinocybe subglobispora*	FFAAS1035	China	OR238887	OR238899	Na et al. (2024)
*Leucoinocybe sulcata*	CAL 1246 (HT)	India	KR029720	KR029721	Deepna Latha et al. (2015)
*Leucoinocybe taniae*	AMB 18838	Italy	OM422766	OM423644	Consiglio et al. (2022)
*Leucoinocybe taniae*	AMB 18839	Italy	OM422767	OM423645	Consiglio et al. (2022)
*Leucoinocybe taniae*	B-4064	Italy	LT854057	LT854028	Antonín et al. (2019)
*Marasmiellomycena albodescendens*	PDD 96321 (HT)	New Zealand	OL998343	OL998382	Consiglio et al. (2022)
*Marasmiellomycena albodescendens*	PDD 96142	New Zealand	OL998341	OL998380	Consiglio et al. (2022)
*Marasmiellomycena tomentosa*	FFAAS1036 (HT)	China	OR238888	OR238900	Na et al. (2024)
*Megacollybia fallax*	DAOM208710	USA	EU623724	-	Hughes et al. (2007)
*Megacollybia fallax*	MICH 45002	USA	EU623714	-	Hughes et al. (2007)
*Megacollybia platyphylla*	BRNM 737654	Czech Republic	LT854048	LT854036	Antonín et al. (2019)
*Mycena purpureofusca*	HMJAU43554	China	LT854049	LT854037	Antonín et al. (2019)
*Mycena purpureofusca*	HMJAU43624	China	MG654741	MK629357	Na and Bau (2018)
*Mycena purpureofusca*	HMJAU43640	China	MG654742	MK629358	Na and Bau (2018)
*Porotheleum fimbriatum*	CLZhao 2368	China	MH114871	-	Direct Submission
*Porotheleum fimbriatum*	Dai 12276	China	KX081137	KX161656	Direct Submission
*Porotheleum parvulum*	JBSD131802 (HT)	Dominican Republic	NR_182714	OM423657	Consiglio et al. (2022)
*Pseudohydropus floccipes*	BRNM 825631	Czech Republic	OM422760	OM423636	Consiglio et al. (2022)
*Pseudohydropus globosporus*	BAP 661 (HT)	USA	OM422758	OM423634	Cooper et al. (2019)
*Pulverulina flavoalba*	FFAAS1039 (HT)	China	OR238891	OR238903	Na et al. (2024)
*Pulverulina flavoalba*	FFAAS1040	China	OR238892	OR238904	Na et al. (2024)
*Trogia benghalensis*	CUH AM122	India	MF967246	-	Dutta et al. (2017)
*Trogia benghalensis*	CUH AM031	India	KU647630	-	Dutta et al. (2017)
*Trogia venenata*	HKAS54710	China	JQ031772	JQ031778	Yang et al. (2012)
*Vizzinia domingense*	JBSD131801a (HT)	Dominican Republic	OM422768	OM423646	Consiglio et al. (2022)
*Vizzinia nigripes*	JBSD131803 (HT)	Dominican Republic	OM422771	OM423648	Consiglio et al. (2022)
*Xuaniella urbica*	HKAS150761 (HT)	China	PX308937	PX309053	Yang et al. (2025)
*Xuaniella urbica*	HTBM2719	China	PX308955	PX309075	Yang et al. (2025)

**Figure 1.**
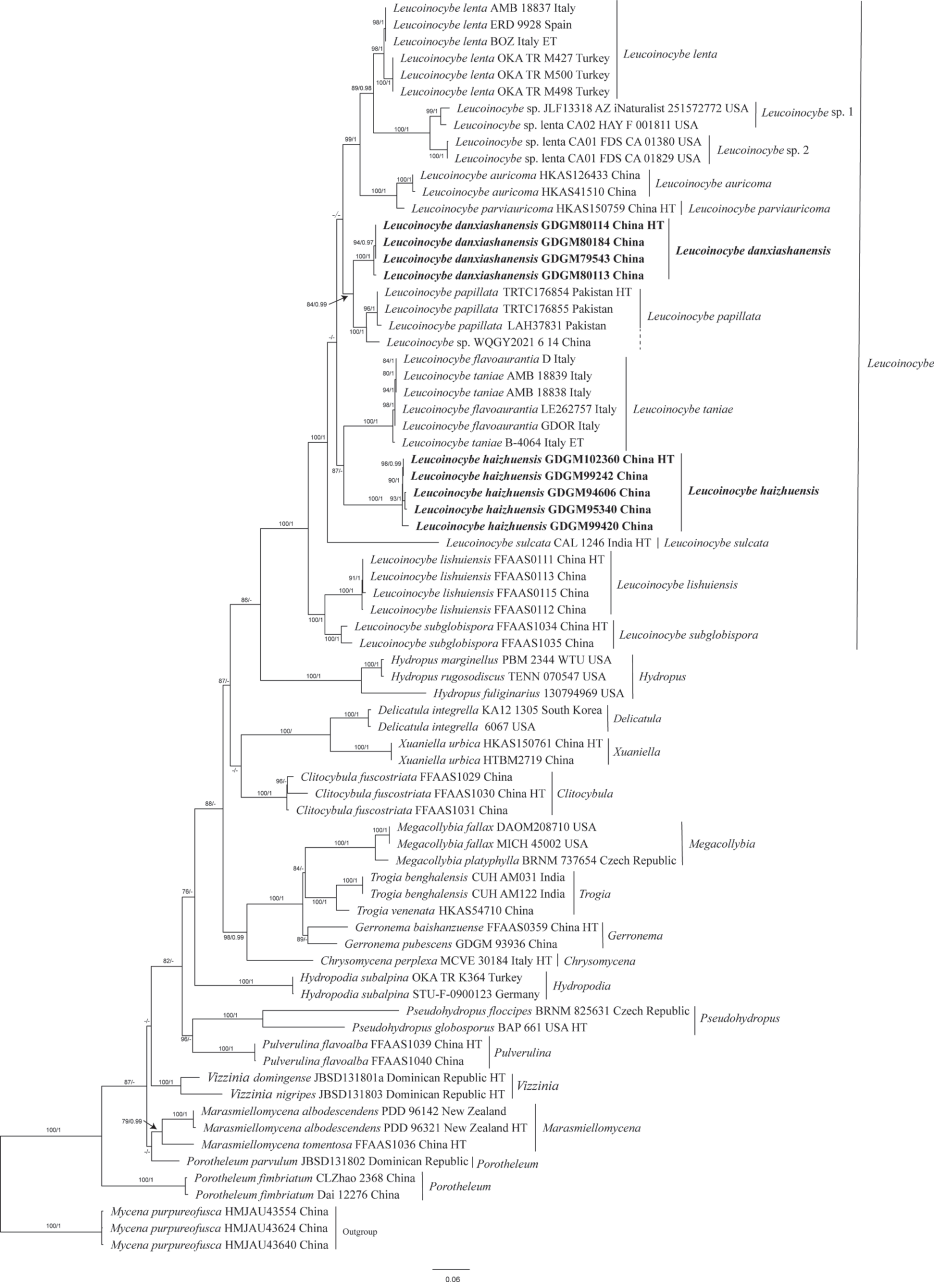
Maximum Likelihood analysis of the genus *Leucoinocybe* based on concatenated nrITS-LSU sequences. Sequences of *Mycena purpureofusca* were selected as outgroups. Nodes were annotated with ML bootstrap ≥ 75% and BPP ≥ 0.95. Holotypes are indicated as HT, and epitypes as ET. New sequences generated in this study were shown in bold.

**Figure 2.**
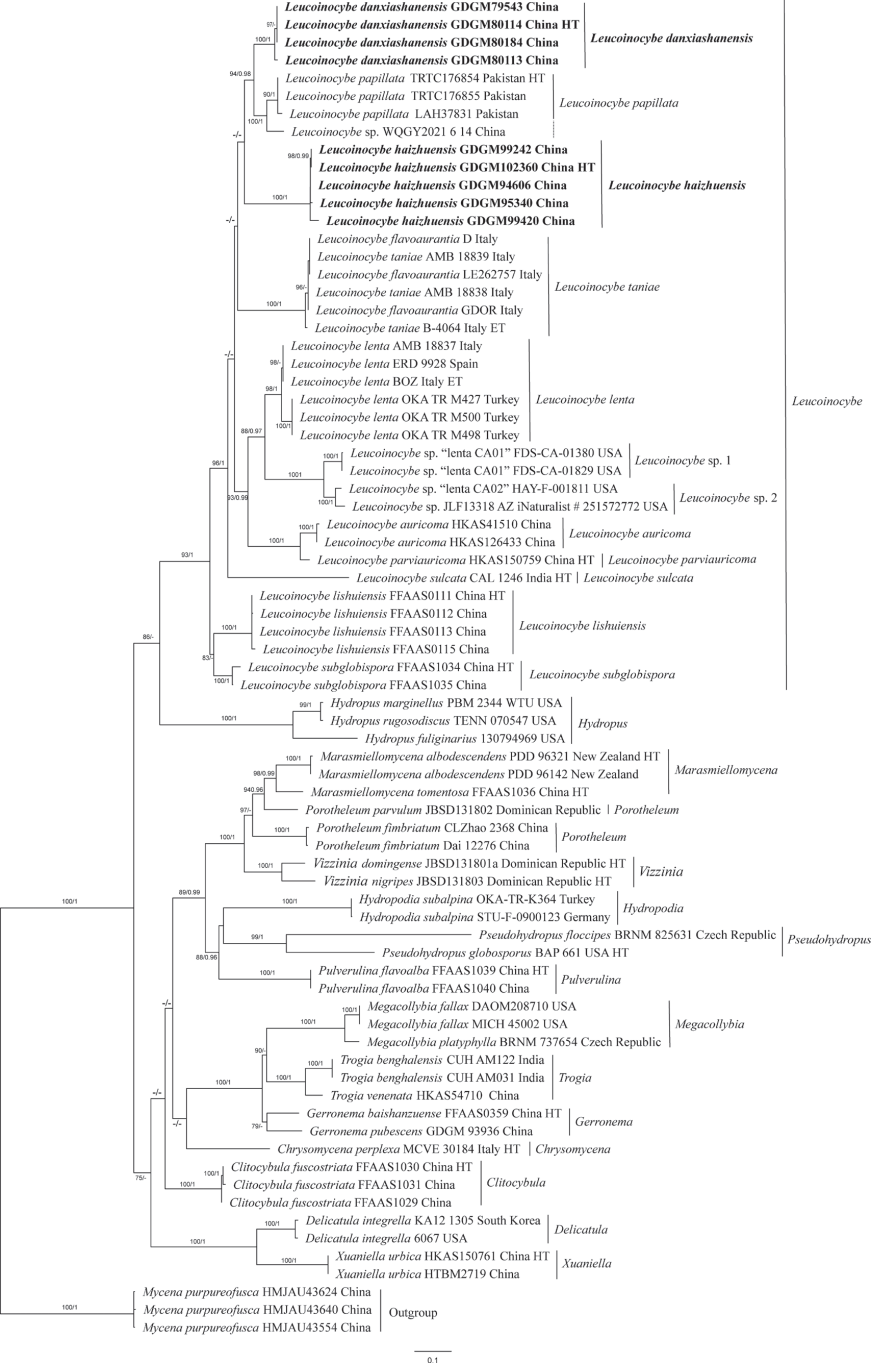
Maximum Likelihood analysis of the genus *Leucoinocybe* based on nrITS sequences. Sequences of *Mycena purpureofusca* were selected as outgroup. Nodes were annotated with ML bootstrap ≥ 75% and BPP ≥ 0.95. Holotypes are indicated as HT, and epitypes as ET. New sequences generated in this study were shown in bold.

The aligned nrITS dataset included 1003 columns, 658 distinct patterns, 104 singleton sites, 418 constant sites, and 481 parsimony-informative characters. The nrITS-LSU dataset had an aligned length of 1784 nucleotide sites including gaps (876 for nrITS and 908 for nrLSU), corresponding to 75 sequences, with 915 constant sites, 652 parsimony-informative characters, 217 singleton sites, and 1020 distinct patterns. For the nrITS dataset, TPM2u+F+I+G4 and HKY+F+I+G4 were selected as the best models for the ML analysis and BI analysis, respectively (Kalyaanamoorthy et al. 2017; Zhang et al. 2020; Xiang et al. 2023). In the nrITS-LSU concatenated dataset, TPM2u+F+I+G4 and TIM2+F+I+G4 were selected as the best models for nrITS and nrLSU separately in ML analysis, and HKY+F+I+G4 and GTR+F+I+G4 were selected as the best models for BI analysis (Kalyaanamoorthy et al. 2017; Zhang et al. 2020; Xiang et al. 2023). The topologies of phylogenetic trees generated from ML and BI analyses were almost identical, thus, only the ML tree’s topologies were shown (Figs 1, 2).

Phylogenetic analyses showed that sequences newly generated from southern China formed two monophyletic lineages within *Leucoinocybe* clade with significantly supported values; additionally, two unnamed lineages existed within *Leucoinocybe*, which were labeled as “*Leucoinocybe* sp. 1” and “*Leucoinocybe* sp. 2” (Figs 1, 2). *Leucoinocybe danxiashanensis* is close to *L. papillata* Aman, Khalid & Moncalvo and a sample labelled as “*Leucoinocybe* sp. WQGY2021-6-14”. While *L. haizhuensis* is sister to *L. taniae* in the nrITS-LSU tree with moderate support value in ML analysis (ML = 90%) (Fig. 1), and formed a distinct lineage in the ITS tree (Fig. 2).

## Taxonomy

### Leucoinocybe danxiashanensis

Taxon classificationFungiAgaricalesPorotheleaceae

Ming Zhang, T.H. Li & Xiang L. Chen sp. nov.

09428AF0-3665-5F5D-9968-2C87DD4066DE

Fungal Names: FN 573015

Figs 3, 4, 5

Chinese name:丹霞山白丝盖伞

**Figure 3.**
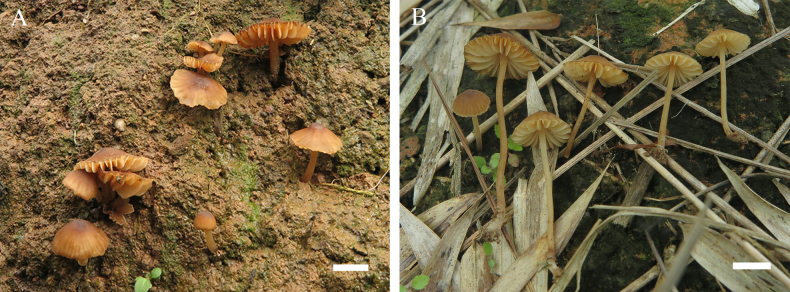
Basidiomata of *Leucoinocybe danxiashanensis*. **A**. Collection GDGM 80114 (holotype); **B**. Collection GDGM 80184. Scale bars: 10 mm (**A, B**).

**Figure 4.**
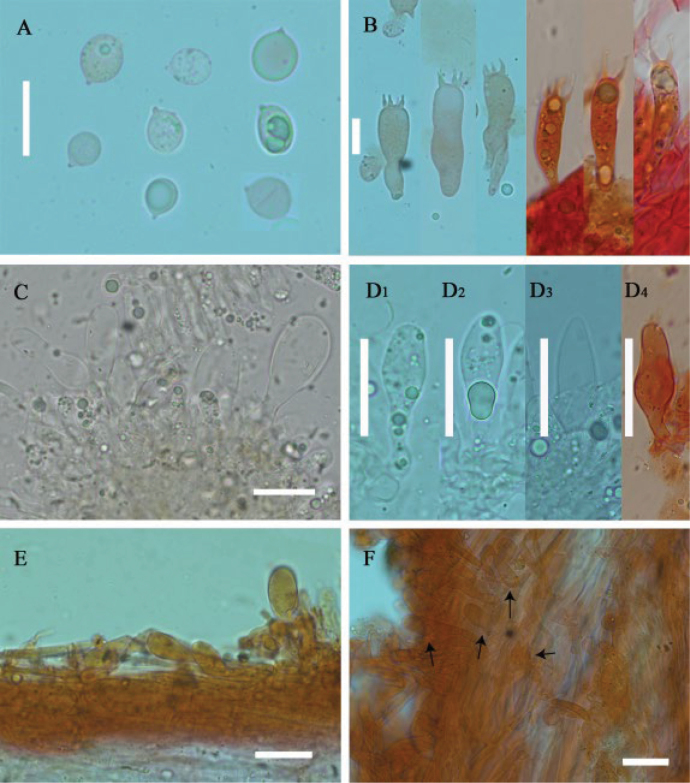
Microscopic features of *Leucoinocybe danxiashanensis* (GDGM 80114). **A**. Basidiospores; **B**. Basidia; **C, D**. Cheilocystidia; **E**. Pileocystidia of pileipellis; **F**. Caulocystidia. The arrows indicate the cystidia. Scale bars: 10 μm (**A, B**); 20 μm (**C–F**).

**Figure 5.**
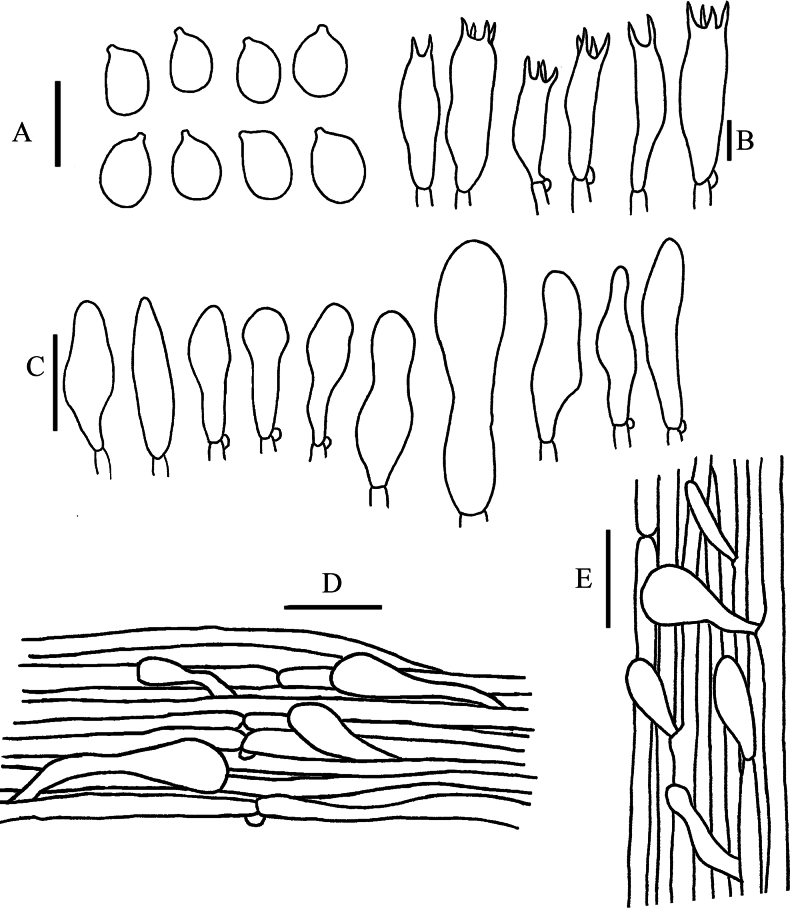
Line drawings of *Leucoinocybe danxiashanensis* (GDGM 80114). **A**. Basidiospores; **B**. Basidia; **C**. Cheilocystidia; **D**. Pileipellis; **E**. Stipitipelli. Scale bars: 10 μm (**A, B**); 20 μm (**C–E**).

#### Diagnosis.

*Leucoinocybe danxiashanensis* is characterized by its mycenoid basidiomata with umbonate at pileus center, sinuate to free lamellae, orange-white pubescence on the stipe surface, diverse cheilocystidia, and broadly elliptical basidiospores.

#### Holotype.

China • Guangdong Province: Shaoguan City, Renhua County, Danxiashan National Nature Reserve, on soil, elev. ca. 150 m, 25°03'N, 113°45'E, 17 September 2015, Ming Zhang (GDGM80114, holotype!).

#### Etymology.

The specific epithet “danxiashanensis” refers to the type locality, the Danxiashan National Nature Reserve in Guangdong Province of China.

#### Description.

***Basidiomata*** small, mycenoid. ***Pileus*** 7–22 mm wide, hemispherical to convex when young, becoming convex to nearly plane when mature, umbonate at center, surface dry, pubescent, smooth or with faint brown-striae at margin, brown (6E7) at center, light brown to brownish orange (6D5–5B4) towards margin; margin straight, slightly undulate. ***Lamellae*** sinuate to free, subdistant to distant, somewhat waxy, white (2A2) to cream when young, orange-white (4A3–5A2) with age, 2–4 mm wide, even at edge, often with 1–3 unequal lamellulae between two entire lamellae. ***Stipe*** 11–40 × 1.5–2.7 mm, central, cylindrical to subcylindrical, straight or curved, yellowish white (4A2) to grayish orange (5B4), cartilaginous, hollow, somewhat translucent, covered with orange-white (5A2) ﬁne pubescence or pruina; basal mycelium white. ***Odor*** and ***taste*** not distinctive.

***Basidiospores*** [40/3/3] 6.5–8.5 × 4.5–6.5 μm, L_m_ = 7.41 ± 0.49 μm, W_m_ = 5.51 ± 0.51 μm, Q = (1.15)1.17–1.45(1.5) μm, Q_m_ = 1.35 ± 0.09, broadly elliptical to elliptical, thin-walled, smooth, guttulate, amyloid. ***Basidia*** 29–40 × 5–7 μm, clavate, hyaline, mostly 4-spored, rarely 2-spored, sterigmata 3–5 μm long, thin-walled. ***Cheilocystidia*** 45–120 × 7–23 µm, scattered, balloon-shaped, broadly conical, narrowly lageniform, narrowly utriform, fusoid or nearly cylindrical, sometimes with long pedicellate, thin-walled, hyaline. ***Pleurocystidia*** rare, similar to cheilocystidia. ***Lamellar trama*** regular to subregular, hyaline hyphae 5–25 μm wide, thin-walled. ***Pileipellis*** a cutis, made up of radially arranged hyaline or slightly pigmented thin-walled hyphae, 3–8 μm wide; ***pileus trama*** interwoven to subregular, hyaline hyphae 14.5–32 μm wide, thin-walled; ***pileocystidia*** 24–60 × 6–9 μm, clavate, fusoid to lageniform, thin-walled to slightly thick-walled, hyaline or pigmented by with brown intraparietal or encrusted pigment. ***Stipitipellis*** a cutis, made up of parallel, repent, thin-walled and hyaline hyphae 2–5 μm wide; ***caulocystidia*** 32–90 × 10–15 µm, numerous, often arising directly from the cutis hyphae, variable in shape, balloon-shaped, clavate, fusoid or lageniform with a long pedicellate neck, thin-walled to slightly thick-walled, hyaline. ***Clamp connections*** present.

#### Habitat and distribution.

Scattered or in small groups on moss bed or humus-rich soil mainly under bamboo groves, elev. 50–150 m, only known from the type locality in southern China.

#### Additional materials examined.

China • Guangdong Province: Shaoguan City, Yinhua Town, Danxiashan National Nature Reserve, on soil, elev. ca. 121 m, 25°01'N, 113°06'E, 22 May 2020, Ming Zhang (GDGM79543); • Same location, elev. ca. 120 m, 25°00'N, 113°68'E, 26 May 2020, Ming Zhang (GDGM80113); Same location, elev. ca. 98 m, 25°00'N, 113°68'E, 28 May 2020, Ming Zhang (GDGM80184).

#### Notes.

Morphologically, *L. danxiashanensis* is similar to *L. sulcata*, they both share the common characters of brownish pileus. However, *L. sulcata* is different in its more distinctly sulcate pileus, subdistant lamellae, glabrous stipe and smaller basidiospores (5–6.5 × 4–5.5 μm) (Deepna Latha et al. 2015). In addition, *L. sulcata* grows on the bark of living *Hydnocarpus* (Flacourtiaceae) trees in the tropical region of India (Deepna Latha et al. 2015).

Phylogenetically (Figs 1, 2), *Leucoinocybe danxiashanensis* is closely related to *L. papillata*, both species have brown pileus and thin-walled cheilocystidia. However, *L. papillata*, originally described from Pakistan, is different from *L. danxiashanensis* in having narrower and off-white lamellae, smaller basidiospores (4.8–7.4 × 3.9–5.8 μm), thick-walled pileocystidia and caulocystidia (Cazabonne et al. 2025).

### Leucoinocybe haizhuensis

Taxon classificationFungiAgaricalesPorotheleaceae

Ming Zhang, C.X. Fan & W.X. Xia sp. nov.

38BDA634-E8B3-5113-9996-526ACAC2CE1A

Fungal Names: FN 573016

Figs 6, 7, 8

Chinese name:海珠白丝盖伞

**Figure 6.**
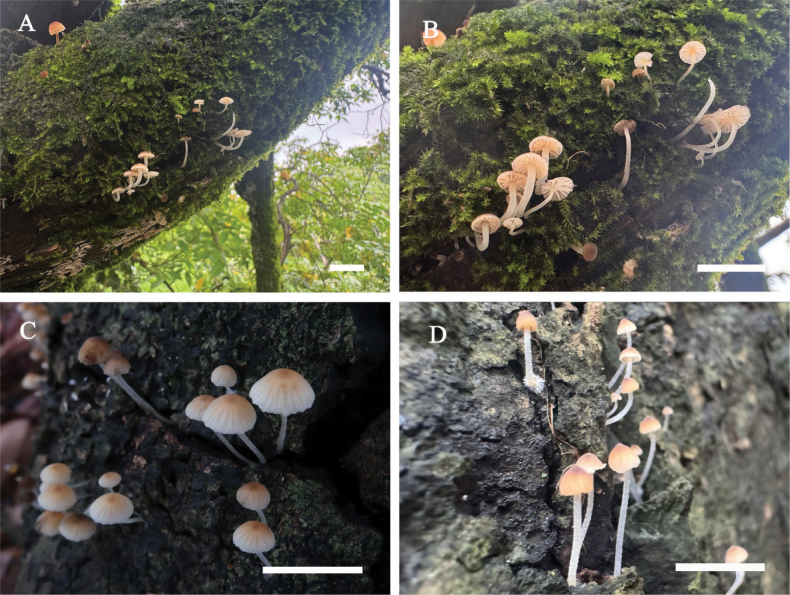
Basidiomata of *Leucoinocybe haizhuensis*. **A, B**. Collection GDGM 102360 (holotype); **C**. Collection GDGM 95340; **D**. Collection GDGM 99420. Scale bars: 10 mm (**A–D**).

**Figure 7.**
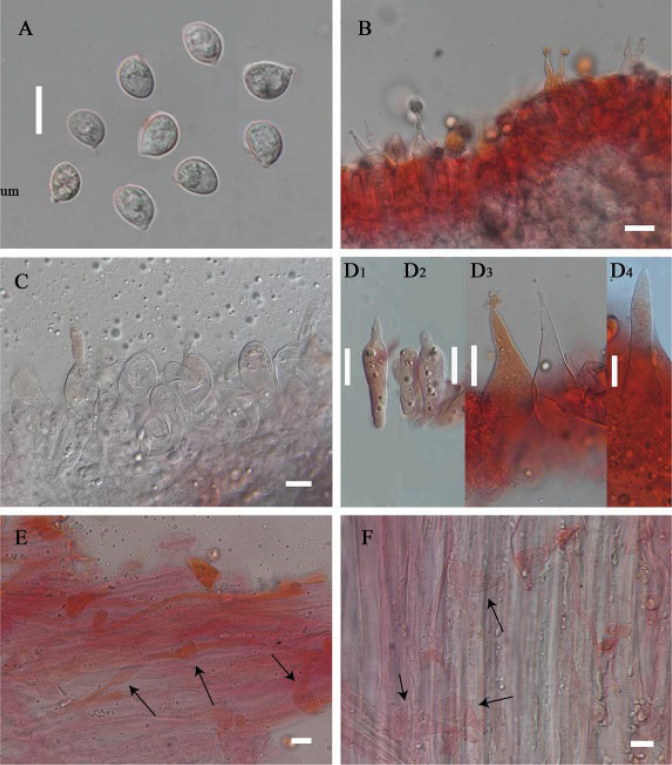
Microscopic features of *Leucoinocybe haizhuensis* (GDGM 102360). **A**. Basidiospores; **B**. Basidia; **C, D**. Cheilocystidia; **E**. Pileocystidia of pileipellis; **F**. Caulocystidia. The arrows indicate cystidia. Scale bars: 10 μm (**A–F**).

**Figure 8.**
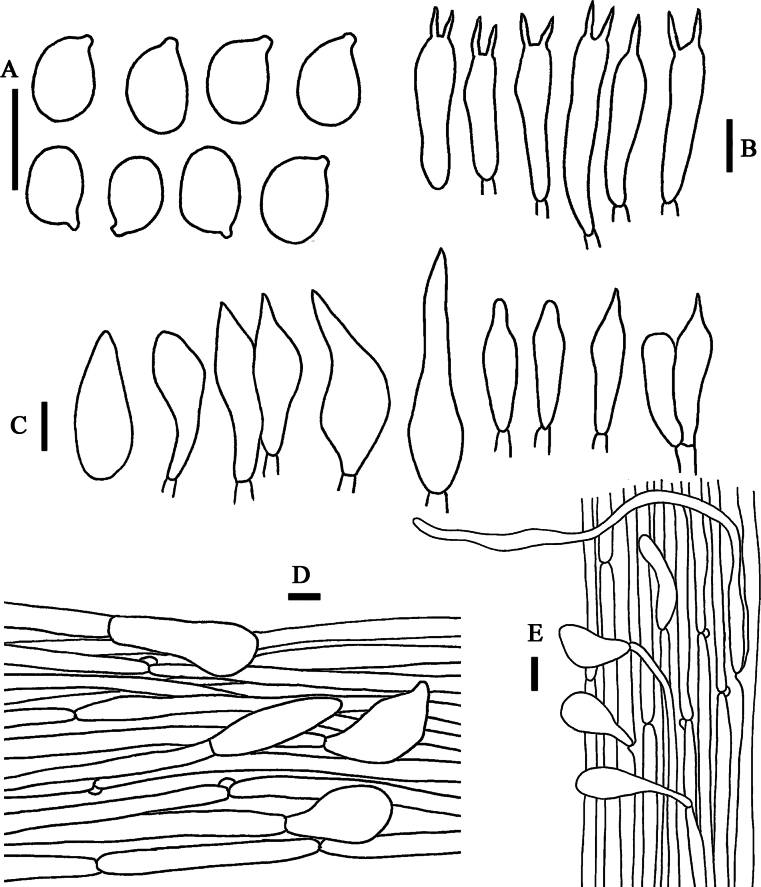
Line drawings of *Leucoinocybe haizhuensis* (GDGM 102360). **A**. Basidiospores; **B**. Basidia; **C**. Cheilocystidia; **D**. Pileipellis; **E**. Stipitipellis. Scale bars: 10 µm (**A–E**).

#### Diagnosis.

*Leucoinocybe haizhuensis* is characterized by its very small basidiomata, brown to yellowish white pileus with finely pubescent on the surface, diverse shaped cheilocystidia, 2-spored basidia, elliptical to broadly elliptical and amyloid basidiospores (7–9 × 5–7 µm).

#### Holotype.

China • Guangdong Province: Guangzhou City, Haizhu National Wetland Park, elev. ca. 4 m, at 23°3'35"N, 113°20'30"E, 7 August 2025, Ruihua Sun and Xinyue Yang (GDGM 102360, holotype!).

#### Etymology.

The specific epithet “haizhuensis” refers to the type locality of this new taxon, the Haizhu Wetland Park in Guangdong Province of China.

#### Description.

***Basidiomata*** small, mycenoid. ***Pileus*** 3–8 mm broad, paraboloid, hemispherical to convex, slightly umbonate at center, surface dry, pruinose, chocolate brown, teak brown, burnt umber (6F6–8), brown (6E4–6), yellowish brown (6D5–8), light orange to pale orange (6B3–5) when young; usually light orange to pale orange (6B3–5) at center, pale orange, orange white to white (5A1–3) toward margin when aged; margin straight, undulate, with light yellow (3A4, 4A4) to pale yellow (3A3, 4A3) striations. ***Lamellae*** adnexed, subdistant to distant, even at edge, white (2A2) to cream, often with 1–3 unequal lamellulae between two entire lamellae. ***Stipe*** 2–25 × 0.3–0.6 mm, central, hollow, subcylindrical, translucent, fragile, chocolate brown, teak brown, burnt umber (6F6–8) to brown (6E4–6) when young, white (1A1) when aged, sometimes with brown (6E4–6) to light orange (5B3–4), obviously light orange (5B3–4) at the base, changing to negro to brownish gray (6F2–3) at age, densely pruinose; basal mycelium white. Odor and taste not recorded.

***Basidiospores*** [50/3/3] 7–9 × (5)5.5–7 μm, L_m_ = 8.23 ± 0.58 µm, W_m_ = 6.24 ± 0.42 µm, Q = (1.15)1.17–1.42(1.45), Q_m_ = 1.32 ± 0.07, broadly elliptical to elliptical, thin-walled, smooth, hyaline in 5% KOH, guttulate, amyloid. ***Basidia*** (18.5)19–35(36.5) × 5.5–7.5(8) μm, clavate, thin-walled, yellowish white to hyaline, 2-spored, with sterigmata 2–7.5 µm long, sometimes with a white patch, clampless. ***Cheilocystidia*** 26–44 × 6.5–13.5 µm, distinct, ovoid, conical, broadly conical, fusiform, broadly fusiform to subcapitate, sometimes apex acuminate to acute, rarely rostrate, thin-walled, hyaline, clampless. ***Pleurocystidia*** absent. ***Lamellar trama*** regular to subregular, hyaline hyphae 12–27.5 μm wide, thin-walled, clamped. ***Pileipellis*** a cutis, composed of fusiform, irregular or subcylindrical hyphae 3–12 μm wide, smooth, thin-walled, yellow to yellowish brown, clamped; ***pileocystidia*** 22–50 × 6.5–21.5 μm, ellipsoid, obovoid, clavate, broadly clavate to utriform, thin-walled to slightly thick-walled, yellowish white to hyaline, clampless. ***Stipitipellis*** a cutis, 3.5–11 μm wide, occasionally upturned, smooth, thin-walled, hyaline, clamped; ***caulocystidia*** 15–147 × 3–8.5 μm, obovoid, clavate, broadly clavate to lanceolate, thin-walled to slightly thick-walled, hyaline, clampless.

#### Habit and distribution.

Scattered or gregarious, living on *Mangifera* trees in broad-leaved forests. Currently only known from the type locality in southwestern China.

#### Additional specimen examined.

China • Guangdong Province: Guangzhou City, Haizhu National Wetland Park, elev. ca. -2 m, at 23°4'39"N, 113°20'4"E, 29 April 2024, Ruihua Sun and Guorui Zhong (GDGM94606); • Same location, elev. ca. 7 m, at 23°3'56"N, 113°20'30"E, 22 August 2024, Ruihua Sun and Liuying An (GDGM95340); • Same location, elev. ca. 7 m, at 23°9'9"N, 113°17'21"E, 19 June 2025, Ruihua Sun and Guorui Zhong (GDGM99242); • Same location, elev. ca. 10 m, at 23°4'48"N, 113°18'12"E, 19 June 2025, Ruihua Sun and Xinyue Yang (GDGM99420).

#### Notes.

Morphologically, *L. lenta* is similar to *L. haizhuensis*. However, *L. lenta* can be distinguished from *L. haizhuensis* by its mostly 4-spored basidia, smaller basidiospores (5.5–8 × 4–5.5 µm), thick-walled and larger pileocystidia (32–140 × 6–11 µm) and caulocystidia (50–150 × 6–16 µm) (Antonín et al. 2019; Kaygusuz et al. 2020; Na et al. 2024). The basidioma color of *L. haizhuensis* is also similar to *L. auricoma*. However, *L. auricoma* differs by its larger basidiomata (pileus up to 25 mm broad), covered with orange to yellowish orange flocci on pileus surface and smaller basidiospores (5–7 × 3–4 µm) (Takahashi 1999).

In nrITS-LSU phylogenetic tree (Fig. 1), *L. haizhuensis* is closely related to *L. taniae*, both species have convex pileus, variably shaped cystidia, and similar-sized basidiospores. However, *L. taniae*, originally described from Spain, is different from *L. haizhuensis* in having larger basidiomata (pileus up to 15 mm broad), orange to reddish brown pileus, creamy or isabelline lamellae, brown to light orange stipe, 4-spored basidia (Vila 2002; Antonín et al. 2019). In nrITS phylogenetic tree (Fig. 2), this new species is placed within *Leucoinocybe* clade, forming a relatively independent clade, and is closely related to *L. danxiashanensis* and *L. papillata*. However, *L. danxiashanensis* is distinguished from *L. haizhuensis* in having larger basidiomata (pileus 7–22 mm broad), light brown to brownish orange pileus, sinuate to free lamellae, narrower basidiospores (6.5–8.5 × 4.5–6.0 µm), and mostly 4-spored basidia; *L. papillata*, originally described from Pakistan, is different from *L. haizhuensis* in having larger basidiomata (pileus up to 16 mm broad), campanulate with a distinct papilla, mustard brown to turning light brown pileus, sinuate lamellae, smaller basidiospores (4.8–7.4 × 3.9–5.8 µm) and absent cystidia (Cazabonne et al. 2025). Besides, *L. danxiashanensis* and *L. papillata* also differ in growing on forest soil, the ecological characteristic that clearly distinguishes them from *L. haizhuensis*.

## Discussion

Phylogenetic analyses (Figs 1, 2) showed that specimens collected from southern China formed two well-supported monophyletic lineages within *Leucoinocybe*, namely, *L. danxiashanensis* and *L. haizhuensis*. Notably, the two new species exhibit distinctly mycenoid basidiomata and the presence of thin-walled pileocystidia and caulocystidia, expanding the known range of cystidia variation within *Leucoinocybe*.

Among the ten known species, seven species are distributed in East and South Asia (Takahashi 1999; Deepna Latha et al. 2015; Na 2019; Na et al. 2021, 2024; Cazabonne et al. 2025; Yang et al. 2025; this study), of which six species have been recorded in China, i.e. *L. auricoma*, *L. danxiashanensis*, *L. haizhuensis*, *L. lishuiensis*, *L. parviauricoma* Kun L. Yang, Jia Y. Lin & Zhu L. Yang and *L. subglobispora* (Na 2019; Na et al. 2021, 2024; Cazabonne et al. 2025; Yang et al. 2025; this study). They are mainly distributed in subtropical or tropical regions, among which, *L. auricoma* can also be discovered in temperate regions (Takahashi 1999; Deepna Latha et al. 2015; Antonín et al. 2019; Na et al. 2021, 2024; Yang et al. 2025). The main distinguishing features of the ten known species of *Leucoinocybe* are summarized in Table 2.

**Table 2.** Currently known *Leucoinocybe* species and their diagnostic characteristics.

Species name	Type location	Pileus	Lamellae	Stipe	Basidiospores	Basidia	Pileocystidia	Caulocystidia	References
*L. auricoma*	Japan	15–25 mm in diam., covered with pale orange to yellowish orange flocci, furfuraceous at center, crenulate pruinose at magrin	free, subdistant, white, with 1–3 lamellae	pale yellow to yellow, covered with yellowish orange flocci, pruinose, without basal mycelium	ovoid-ellipsoid to elliptical, 5–7 × 3–4 µm, smooth, thin-walled, amyloid	4-spored	thick-walled	thick-walled	Takahashi 1999; Na et al. 2019
*L. danxiashanensis*	China	7–22 mm in diam., brown at center, light brown to orange at margin, pubescent	sinuate to free, subdistant to distant, pale cream to orange white, with 1–2 lamellae	yellowish white to greyish orange, covered with orange white ﬁne pubescence or pruina, basal mycelium white	broadly elliptical to subamygdaliform, 6.5–8.5 × 4.5–6 µm, smooth, thin-walled, amyloid	2 or 4-spored	thin-walld to slightly thick-walled	thin-walld to slightly thick-walled	This study
*L. haizhuensis*	China	3–10 mm in diam., brown to orange at center, orange to white at margin, pruinose	adnexed, subdistant to distant, yellowish white to white, with 1–3 lamellae	brown, yellowish wihte to white, sometimes light brown at the base when old, pruinose, basal mycelium white	elliptical to broadly elliptical, 7–9 × 5–7 µm, smooth, thin-walled, amyloid	2-spored	thin-walld to slightly thick-walled	thin-walld to slightly thick-walled	This study
*L. lishuiensis*	China	2.5–13.5 mm in diam., pale brown to brown, pruinose at center	adnexed to slightly decurrent, white, with 1–2 lamellae	white, light brown at the base when old, densely pruinose, basal mycelium white	narrowly elliptical, 7.9–10.7 × 4.2–6.1 µm, smooth, thin-walled, amyloid	2 or 4-spored	thick-walled in the middle part and with a thin-walled base	thick-walled in the middle part and with a thin-walled base	Na et al. 2021
*L. lenta*	Italy	up to 60 mm in diam., brown to reddish ochre or pale pinkish-beige at the centre, fibrillose	free, ventricose and unequal, distant, white to cream	whitish with a brownish base, rooting, pruinose	broadly ellipsoid to ellipsoid, 5.5–8.0 × 4.0–5.5 µm, smooth, thin-walled, amyloid	4-, rarely 2-spored	thick-walled	thick-walled	Gröger 2006; Eyssartier and Roux 2011; Antonín et al. 2019; Kaygusuz et al. 2020
*L. papillata*	Pakistan	2.6-16 mm in diam., brown light brown, granular pileus surface	sinuate, off-white	white	ellipsoid, 4.8–7.4 × 3.9–5.8 µm, smooth, amyloid	4-spored	-	thick-walled	Cazabonne et al. 2025
*L. parviauricoma*	China	3 mm in diam., bread orange to honey or ange	free, crowded, light cream orange	subtranspar ently whitish at background, covered with furfuraceous, bread orange to honey orange squamules	elliptical, 4.5–5 × 3–3.5 µm, thin-walled, smooth, nearly colorless, amyloid	2, 3 or 4-spored	absent	thick-walled	Yang et al. 2025
*L. subglobispora*	China	2.5–8 mm in diam., dark brown to light smoke gray, pruinose	adnexed to slightly decurrent, white, with 1–2 lamellae	white, light olive yellow at the base when old, densely pruinose, basal mycelium white	subglobose to broadly elliptical, 5.6–7.5 × 4.8–6.8 µm, smooth, thin-walled, amyloid	4-spored	thick-walled	thick-walled	Na et al. 2024
*L. sulcata*	India	13–52 mm in diam., light brown, brown to grayish orange, granulose	sinuate, transveno-se, subdistant, brownish orange or brownish yellow, with 2–3 lamellae	brownish yellow or golden brown, sparsely pruinose	broadly ellipsoid to subamygdaliform, 5–6.5 × 4–5.5 µm, smooth, thin-walled, amyloid	4-spored	absent	absent	Deepna Latha et al. 2015
*L. taniae*	Spain	15 mm in diam., orange to reddish brown, pruinose-fibrillose	decurrent, cream to pale beige	light orange to brown, pruinose	elliptical to subglobose, 7.5–9 × 5.5–6.5 µm, thin-walled, smooth amyloid	4-spored	thin-walld	thin-walld	Vila 2002; Antonin et al. 2019

## Supplementary Material

XML Treatment for Leucoinocybe danxiashanensis

XML Treatment for Leucoinocybe haizhuensis

## Additional information

### Use of AI

No use of AI was reported.

### Author ORCIDs

Wen-Xiao Xia https://orcid.org/0009-0006-0483-205X

Chao-Qun Wang https://orcid.org/0000-0001-8665-0618

Xiang-Lian Chen https://orcid.org/0000-0002-2668-5840

Rui-Hua Sun https://orcid.org/0009-0002-4559-4669

Cun-Xiang Fan https://orcid.org/0009-0005-1833-6842

Tai-Hui Li https://orcid.org/0000-0002-7343-0365

Wang-Qiu Deng https://orcid.org/0000-0001-8790-2549

Hai-Mei Yue https://orcid.org/0000-0001-9282-2295

Ming Zhang https://orcid.org/0000-0001-6420-2531

### Data availability

All of the data that support the findings of this study are available in the main text.
